# Degradation Kinetics of Clavulanic Acid in Fermentation Broths at Low Temperatures

**DOI:** 10.3390/antibiotics8010006

**Published:** 2019-01-17

**Authors:** David Gómez-Ríos, Howard Ramírez-Malule, Peter Neubauer, Stefan Junne, Rigoberto Ríos-Estepa

**Affiliations:** 1 Grupo de Bioprocesos, Departamento de Ingeniería Química, Universidad de Antioquia UdeA, Calle 70 No. 52-21, Medellín 050010, Colombia; rigoberto.rios@udea.edu.co; 2 Escuela de Ingeniería Química, Universidad del Valle, A.A. 25360, Cali 76001, Colombia; 3 Chair of Bioprocess Engineering, Institute of Biotechnology, Technische Universität Berlin, Ackerstr. 76, ACK 24, 13355 Berlin, Germany; peter.neubauer@tu-berlin.de (P.N.); stefan.junne@tu-berlin.de (S.J.)

**Keywords:** clavulanic acid, degradation, kinetics, equilibrium, irreversible, imidazole, reaction mechanism, fermentation broth, low temperature

## Abstract

Clavulanic acid (CA) is a β-lactam antibiotic inhibitor of β-lactamase enzymes, which confers resistance to bacteria against several antibiotics. CA is produced in submerged cultures by the filamentous Gram-positive bacterium *Streptomyces clavuligerus*; yield and downstream process are compromised by a degradation phenomenon, which is not yet completely elucidated. In this contribution, a study of degradation kinetics of CA at low temperatures (−80, −20, 4, and 25 °C) and pH 6.8 in chemically-defined fermentation broths is presented. Samples of CA in the fermentation broths showed a fast decline of concentration during the first 5 h followed by a slower, but stable, reaction rate in the subsequent hours. A reversible-irreversible kinetic model was applied to explain the degradation rate of CA, its dependence on temperature and concentration. Kinetic parameters for the equilibrium and irreversible reactions were calculated and the proposed kinetic model was validated with experimental data of CA degradation ranging 16.3 mg/L to 127.0 mg/L. Degradation of the chromophore CA-imidazole, which is commonly used for quantifications by High Performance Liquid Chromatography, was also studied at 4 °C and 25 °C, showing a rapid rate of degradation according to irreversible first-order kinetics. A hydrolysis reaction mechanism is proposed as the cause of CA-imidazole loss in aqueous solutions.

## 1. Introduction

Clavulanic acid (CA) is a soft β-lactam antibiotic with a strong inhibitory effect on β-lactamase enzymes, which confer resistance to bacteria against several known broad-spectrum antibiotics. CA is produced by the filamentous Gram-positive bacterium *Streptomyces clavuligerus (S. clavuligerus)* as secondary metabolite related to the clavams pathway derived from arginine and glycerol metabolism [1,2]. The *Streptomyces* genus is a typical source of essential antimicrobial compounds; their production is commonly carried out in liquid cultures where expression of secondary metabolite mycelia occurs [3,4]. CA production is usually performed in submerged cultures of *S. clavuligerus* under aerobic conditions with glycerol as carbon source, maintaining controlled conditions of pH and temperature [5,6,7,8,9]. Previous studies have shown that CA stability increases at slightly acidic pH values [10,11]; several authors found a pH of 6.8 as favorable for obtaining high titers of CA in submerged cultures [5,7,9,12,13,14,15,16].

CA chemical instability largely depends on the pH-value owing to the presence of a carbonyl group linked to the β-lactam ring, which is susceptible to acid or basic catalyzed water attacks [17]. CA is also susceptible to moderate temperature increments, since they accelerate the rate of degradation regardless of the source [10]. Ishida et al. [18] showed that CA is unstable in production media, which contains ammonium ions and amine groups due to the presence of ammonium salts and amino acids. 

Bersanetti et al. [10] investigated CA degradation at 10, 20, 25, 30, and 40 °C and pH values of 6.2 and 7.0. The results fitted an irreversible first-order kinetics accounting for the relationship between the degradation rate constant and the temperature. The highest CA stability was found at slightly acid conditions (pH 6.2) and low temperatures (10 °C). It was also observed that CA which originated from fermentation broth, degraded faster than the pure reagent and the commercial medicine [10]. 

The decomposition kinetics of CA in concentrations between 2.5 and 20 g/L was investigated by Brethauer et al [19]. A first-order kinetic model was proposed where the kinetic constant increased while increasing the initial CA concentration, indicating that CA accelerated its own decomposition [19]. Carvalho et al. [17] explored the CA long-term stability under different conditions of pH (4.0–8.0), temperature (20–45 °C) and ionic strength. The optimal conditions for achieving a low degradation rate were pH 6.0–7.2 and 20 °C; in contrast, addition of inorganic salts (NaCl, Na_2_SO_4_, CaCl_2_, and MgSO_4_) increased instability of CA, possibly due to the higher rate of collisions between molecules within the solution [17]. 

Marques et al. [11] studied the thermal effect on CA production during fermentation in the temperature range from 24 to 40 °C. The highest rates of CA formation and degradation were observed at relatively high temperatures (32 and 40 °C). The course of CA concentration fitted irreversible first-order kinetics and the temperature dependence followed an Arrhenius-type behavior [11].

Similarly, Costa and Badino [16] investigated the impact of temperature reduction from 30 to 25 °C, 30 to 20 °C, and 25 to 20 °C on CA titers during cultivation. The authors observed that temperature reduction had a stronger impact on CA production rather than on the reduction of CA degradation [16].

Different hypotheses regarding CA degradation have been proposed. It has been reported that amino acids and other metabolites or compounds present in the culture media affect the CA degradation, their effect is attributed to the polar groups probably attacking the four-ring lactam carboxyl group of CA to open the β-lactam ring [18,20]. The β-lactam compounds are susceptible to acid–base catalysis and contain groups such as amino or hydroxyl, which can act as catalysts leading to a self-catalyzed decomposition [19].

Due to the wide use of CA in pharmaceutical industry, its production is an intensive field of research [1]. CA stability in fermentation broths is of interest, especially for downstream processing, analysis, conservation and storage. Kinetics of CA degradation in fermentation broths at low temperatures have not been explored so far. This contribution is aimed at modeling and studying the kinetics of CA degradation at low temperatures (−80, −20, 4, and 25 °C) and pH 6.8 in chemically defined fermentation broths, as well as during the imidazole–derivatized conditions.

## 2. Results and Discussion

### 2.1. Clavulanic Acid Degradation

**Figure 1:**
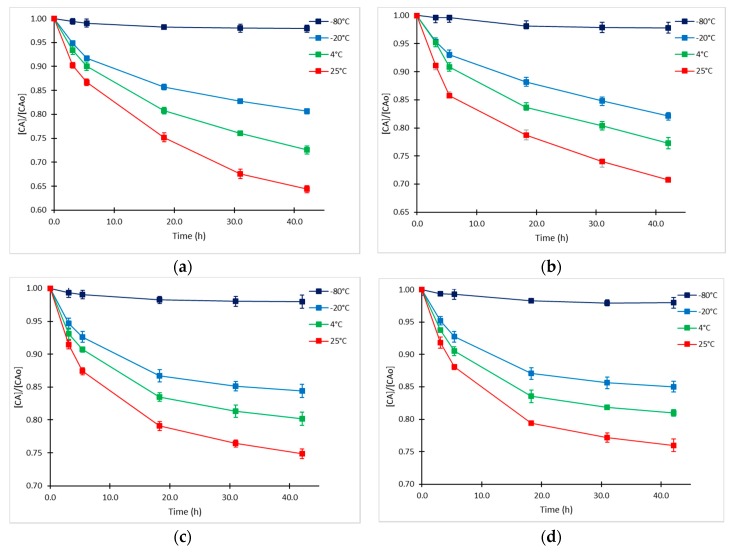
Time courses of relative CA concentration at −80, −20, 4 and 25°C and different CA initial concentrations (CA_o_). (**a**) CA_o_= 126.7 mg/L; (**b**) CA_o_= 65.5 mg/L; (**c**) CA_o_= 25.3 mg/L; and (**d**) CA_o_= 16.3 mg/L.

Previous studies have shown the susceptibility of CA to be decomposed in solution and fermentation broths when temperature ranges from 10 °C to 40 °C [10,11,12]. As far as we know, kinetics of CA degradation in fermentation broths at low temperatures have not been explored. The initial CA concentrations (CA_o_) in supernatant samples included in the experimental design were 65.48 ± 0.04 mg/L (high level), 25.29 ± 0.03 mg/L (medium level), and 16.33 ± 0.04 mg/L (low level). An additional sample of higher concentration (CA_o_ =126.67 ± 0.04 mg/L) was included in the study. The experimental data presented in Figure 1 showed that the degradation proceeded at the highest rate during the first 5–6 h. Product loss was between 8% and 12% during this time. Interestingly, the rate of decomposition tended to slow down markedly as CA_o_ decreased, which was also confirmed in the statistical analysis. As expected, the degradation of CA was considerably accelerated when temperature increased; thus, at the highest temperature of exposition (25 °C), the accumulated loss of product reached 35% at 42 h at the highest initial CA_o_. Comparable results were obtained by Ishida et al. [18] and Roubos et al. [20] in fermentation broths at 28°C with similar composition. In contrast, at the lowest temperature that was evaluated (−80 °C), the exhibited degradation rate was minimum, since less than 4% was lost in 42 h. This is a desirable condition for long-term conservation of supernatant fermentation samples, assuring a stability of the product of interest. 

The high degradation rate at −20 °C was not initially expected; however, similar behavior was observed for other beta lactam antibiotics in aqueous solutions and CA in its pharmaceutical form [21,22,23]. This behavior might be caused by the expulsion of the solute to the liquid portion of the mixture, which can form a layer on the surface of ice or can be trapped between growing ice grain [24]. Thus, CA degradation would continue in the liquid portions. Additionally, given the continuous reduction of the liquid volume, the local concentration of the solute in this limited volume increases significantly also increasing the reaction rate [24]. Nevertheless, this condition must not remain for a long time since complete crystallization of the solution will necessarily stop the reaction. This condition might also explain the trends of degradation at −80 °C and its stabilization beyond 20 h. 

**Figure 2:**
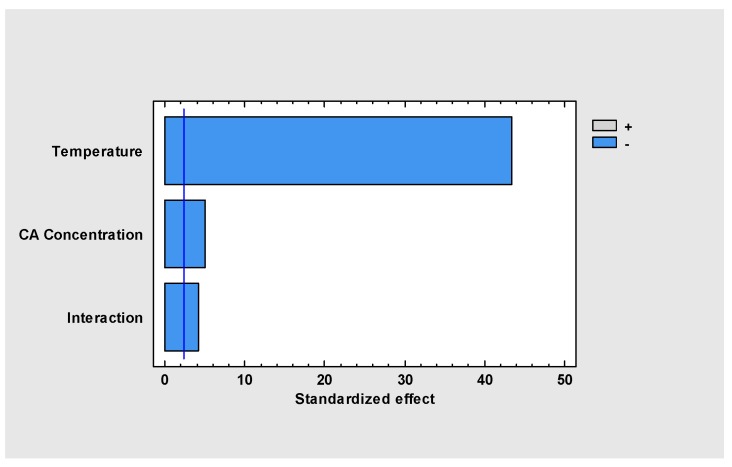
Temperature and concentration effects on CA final concentration.

The statistical analysis with 95% of confidence (r_2_ = 0.996) indicates that both, temperature and CA_o_, affect the degradation of CA in the time range explored (0 to 42.1 h.). The Pareto standardized chart (Figure 2) represents graphically the analysis of variance, showing the significant effects on the response variable, i.e., final CA concentration. The results of the analysis of variance show that the temperature effect is much more significant than CA_o_ and the combined interaction effect, influencing the final CA concentration negatively in the period between 0 and 42 h. 

The trends of CA decomposition in fermentation broth seem to deviate from first and pseudo-first-order kinetics previously reported for CA solutions prepared with standard reactant or commercial formulations [10,19,23,25,26,27]. Similar behaviors to those observed in this work can be observed in the data presented by other authors for CA from fermentation broths in the range of 10 to 40 °C [10,17]. The rate of CA degradation is susceptible to medium composition, exhibiting higher degradation rates in supplemented and complex media [10,11,16]. In the present study, the degradation rate was expected to be low since the substances present in the supernatants would be considerably less. 

**Figure 3:**
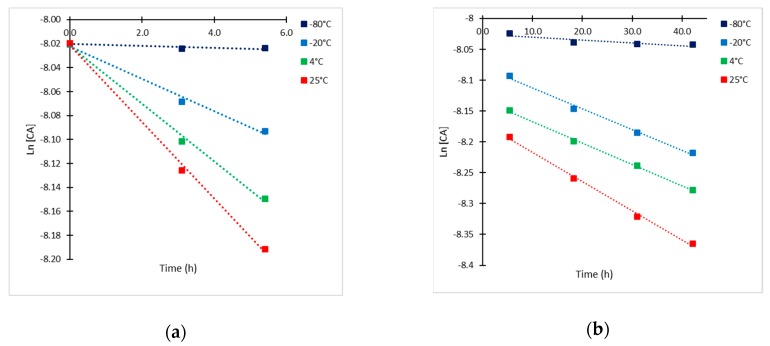
Semi-log plots of CA concentration (CA_o_ = 65.5 mg/L) at −80, −20, 4 and 25 °C. (**a**) t ≤ 6 h; and (**b**) t > 6 h.

Due to experimental limitations regarding the analysis of the products of CA decomposition, only the time course of CA concentration could be properly followed. Initial CA concentration of 65.5 mg/L was chosen as the base case for the estimation of the kinetic parameters of degradation for being in the middle of the range of concentrations under study. In Figure 3, a semi-log representation of CA at different temperatures is presented. A more pronounced slope of the line is attained during the first 5–6 h of exposition, the slope decreased substantially thereafter. Thus, two kinetic constants (k_obs,1_ and k_obs,2_) were determined by means of a linear regression from data plotted in Figure 3. The results of the observed rate constants k_obs,1_ for t < 5.5 h and k_obs,2_ for t > 5.5 h are presented in Table 1.

### 2.2. Kinetic Approach of CA Degradation

The increase of the reaction rate with temperature rise is typical of Arrhenius type kinetics and the significant effect of CA_o_ can be attributed to self-catalysis [19] or degradation reaction with stoichiometric coefficient higher than one. In this case, the dynamics of CA concentration at different temperatures do not fit with irreversible first-order or pseudo-first-order models; rather they are similar to those observed in two consecutive equilibrium-irreversible first-order reactions (Equations (1) and (3)). A similar approach was also proposed by Carvalho et al. [17] for degradation of CA solutions prepared with standard reactant, at temperatures ranging from 20 to 40 °C. The observed time courses of CA degradation are typical of consecutive equilibrium-irreversible reactions in which the reaction rate of the irreversible reaction is considerably low in comparison to the equilibrium reaction (i.e., k_2_ < k_1_ < k_−1_) [17]. Under these conditions, irreversible reaction would be the rate-limiting step, hence the reactant and the intermediate would be in equilibrium condition. Based on this analysis, the following kinetic mechanism of CA degradation was considered: a first step occurs in equilibrium, in which CA produces an active intermediate (I*), hereinafter this intermediate reacts irreversibly with an additional CA molecule to form the degradation product (D).

(1) $${\mathrm{CA}⇆I}^{*}\;r_{1}=k_{1}\left[\mathrm{CA}\right]\;-k_{-1}\left[I^{*}\right]\;$$

(2) $$K_{\mathrm{eq}}=\frac{{\;k}_{1}}{k_{-1}}$$

(3) $$I^{*}+\;\mathrm{CA}\to D\;r_{2}\;={\;k}_{2}\left[\mathrm{CA}\right]\;$$

**Figure 4:**
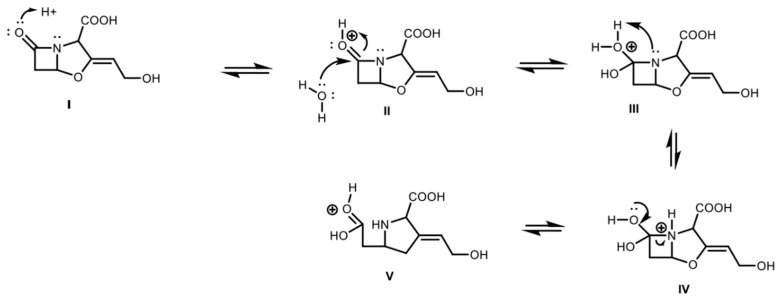
Early steps of CA degradation: (I). Protonation of carbonyl group in the β-lactam ring. (II). Nucleophilic attack of water to carbonyl group. (III). Deprotonation of water–protonation of the nitrogen. (IV–V). Breaking of C–N bond in the β-lactam ring.

This kinetic approach is consequent with a feasible chemical mechanism of reaction for CA in aqueous solutions, characterized by several equilibrium steps (Figure 4) [28,29]. Additionally, this molecule has different potential candidate nodes (C atoms bonded to N and/or O) for suffering nucleophilic attacks from the substances present in the medium, e.g., CA itself, amino acids, and other metabolites with electronegative groups. In this regard, Zhong et al. [30] proposed a mechanism of formation of the degradation product known as substance E; this mechanism involves several steps, including the irreversible reaction of an active intermediate with an additional molecule of CA leading to the reported decomposition product. 

In the general case, the early steps of CA degradation must involve the equilibrium reaction of opening of the β-lactam ring, via protonation of oxygen and nucleophilic attack of a water molecule to the carbonyl group (Figure 4). Once the open intermediate is formed, several irreversible possibilities can occur like nucleophilic attacks by nitrogen on other CA molecules or amino acids, imine formation via decarboxylation or attack of other substances present in the medium [31].

Under this kinetic approach, two kinetic rate constants were determined from experimental data: the equilibrium constant (K_eq_) and the irreversible rate constant (k_2_). During the first 5–6 h of exposition, and according to Equation (1), the equilibrium reaction is favoring the formation of intermediate I*, which is converted irreversibly into product D at lower reaction rate, thus the irreversible reaction (Equation (2)) is the rate-limiting step. This condition, allowed to determine the rate constant (k_2_) for the irreversible reaction from semi-log plot of CA concentration at each temperature (Figure 3a) [17]. Values of the irreversible rate constant (k_2_) were previously determined as k_obs,1_. 

The significant concentration of intermediate I* after 6 shifts the equilibrium towards CA and hence, the equilibrium reaction (Equation (1)) is the rate-limiting step [17]. In the case of the equilibrium constant (K_eq_), values were determined from experimental time courses of CA concentration by applying parameter estimation using the Levenberg-Marquadt method for least squares minimization. The summary of kinetic parameters for the two-reaction model for CA degradation is presented in Table 2. 

**Figure 5:**
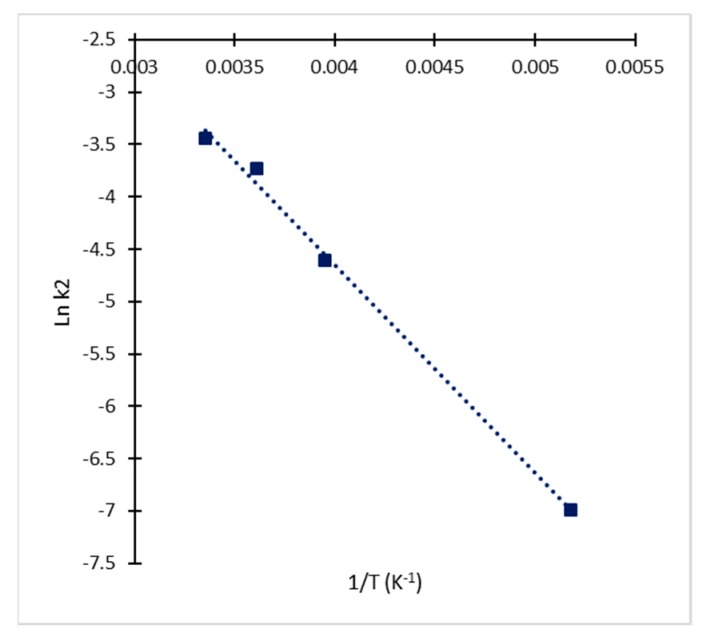
Arrhenius plot for determination of kinetic parameters (E_a_ and A) for the irreversible reaction of CA degradation.

The estimated values of equilibrium constant confirmed the initial assumption of k_2_ < k_1_ < k_−1_, since all the calculated values for k_2_ were lower than K_eq_, and all the estimated values of K_eq_ were lower than 1. For the case of the irreversible reaction (Equation (3)), the dependence of rate constant k_2_ on temperature and activation energy were determined through linear regression of data in the Arrhenius plot (Figure 5). The activation energy (E_a_) calculated for the irreversible process of degradation was 16.512 kJ/mol which is close to previous reports for CA in fermentation broths [10,17]. The corresponding frequency factor (A) was 26.97 h^−1^ with a correlation coefficient (r^2^) of 0.961. The standard Gibbs free energy of activation for the reversible reaction (ΔG°) was determined assuming the previously determined equilibrium at 25 °C as the standard condition yielding a value of 3.155 kJ/mol. This shows that decomposition via a transition state is thermally activated. Marques et al. [11] pointed out that ΔG° values for CA formation are slightly higher than those for CA degradation and at moderate temperatures the degradation of CA would be practically unavoidable. This fact explains the accumulation of CA in the fermentation broth while the microorganism is active despite the continuous degradation. Yet, when the production rate decreases, CA would eventually disappear from the broth due to decomposition. 

### 2.3. Kinetic Model Validation

**Figure 6:**
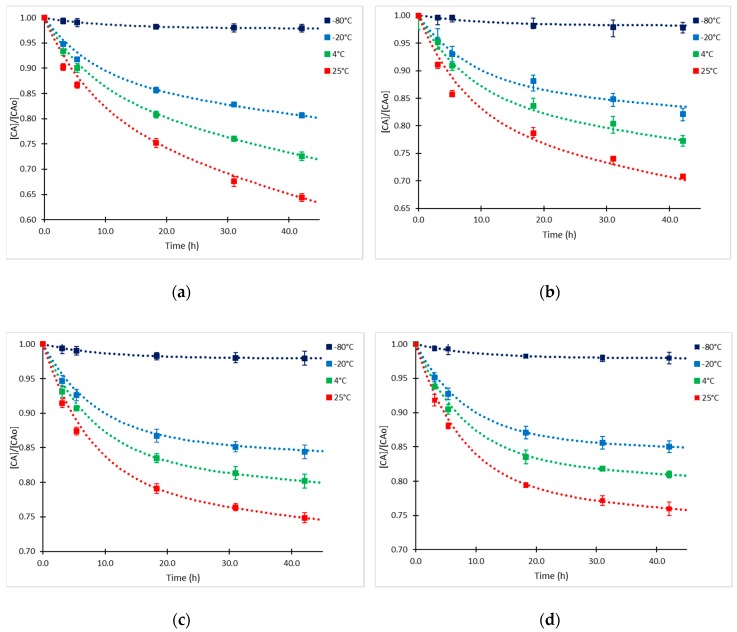
Experimental points (squares) and model prediction (dashed line) of CA degradation at −80, −20, 4 and 25 °C. (**a**) CA_o_= 126.7 mg/L; (**b**) CA_o_= 65.5 mg/L; (**c**) CA_o_= 25.3 mg/L; and (**d**) CA_o_= 16.3 mg/L.

The proposed kinetic approach was validated with all the experimental data of CA degradation obtained in this study. Numerical simulations of the kinetic model were performed, and their results were compared with the experimental results of CA degradation at all temperatures and CA_o_ values. The CA relative concentration time courses and the corresponding experimental points are presented in Figure 6. The normalized-root-mean-square error (NRMSE) calculated between the complete experimental dataset and model predictions was used for assessing the accuracy of the kinetic model (Table 3).

As observed in Figure 6, the simulated time course of CA concentration shows a good approximation to the experimental values, notwithstanding the assumptions made for the determination of kinetic parameters. The proposed model of degradation simplified the decomposition of CA to only one intermediate product and one decomposition product following equilibrium and irreversible first-order kinetics; hence, deviations were expected between experimental and simulated data. Nevertheless, in the range of the concentrations and temperatures evaluated, the model showed a good fit, since the deviation of the predicted and measured concentrations was less than 5% in all cases. As it can be observed in Table 3, under this kinetic mechanism, the shelf-life of the product is highly dependent on temperature and initial CA concentration and not only on the rate constant as the case of the irreversible first-order reactions. The results obtained for the shelf-lives of CA in fermentation broth are comparable to the data obtained by Jerzsele and Nagy [26] for aqueous solutions of standard clavulanate, Bersanetti et al. [10], Ishida et al. [18] and Roubos et al. [20] for CA degradation in fermentation broths at room temperature. The shelf lives obtained at −20 °C confirm the significant degradation rate at this temperature, similar observations were reported in the temperature range from −7 to −25 °C for CA solutions of pharmaceutical CA [23] and other β-lactam compounds [21,22]. In all cases, the shelf lives of the CA in supernatants are considerably lower than those reported for CA aqueous solutions prepared with pharmaceutical formulations [24,25,32], possibly due to the addition of some stabilizing components to assure a long life in the commercial product.

The proposed kinetic approach for CA degradation explains the observed effect of increase in the reaction rate with increasing CA_o_. The equilibrium-irreversible reaction model fits not only the hypothetical chemical reaction mechanisms involving equilibrium steps, but also the potential enzymatic degradation of CA in fermentation broths at temperatures higher than 4 °C, since Michaelis-Menten kinetics represents a special case of this kind of reactions. However, the most significant effect on CA degradation seems to be the chemical mechanism, considering the rapid degradation also observed in standard solutions and (aqueous) pharmaceutical products, where the presence of enzymes or proteins is discarded. Moreover, Ishida et al. [18] found no influence of exocellular proteins (in the culture broth) on the degradation of CA; hence, the enzymatic effect would not be considered as a degradation factor. Presence of free amino acids and decomposition products in CA solutions increase the degradation rate [18,19]; this might be an indicator of susceptibility of CA molecules to nucleophilic attacks added to the variable catalytic effect of pH [10,17,23,25]. 

### 2.4. Derivatized Clavulanate-Imidazole Degradation Kinetics

**Figure 7:**
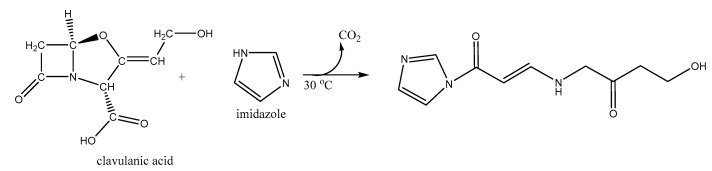
Derivatization of CA with imidazole: Alkylation of secondary nitrogen in the imidazole.

CA is poorly retained in C-18 reverse-phase columns for High Performance Liquid Chromatography (HPLC) and it does not produce distinctive peaks. Therefore, derivatization of CA is required to generate the chromophore clavulanate-imidazole (CAI), which is detectable at a wavelength of 311 nm. During derivatization the β-lactam ring of CA is opened, yielding decarboxylation and formation of an amido group as shown in Figure 7.

**Figure 8:**
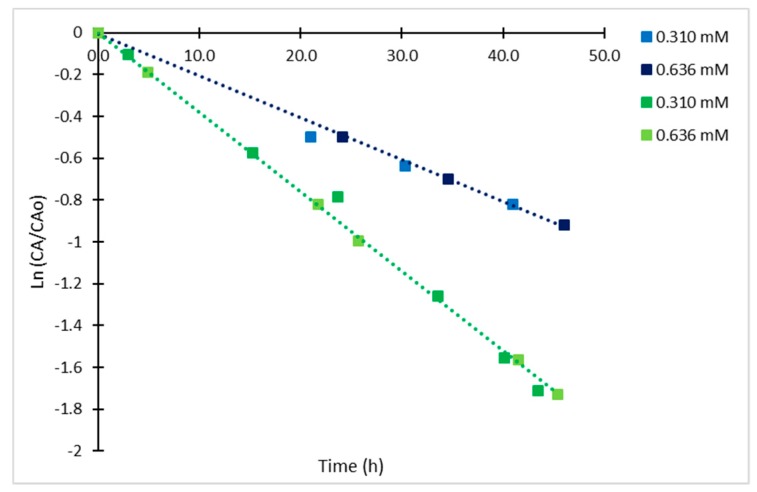
Semi-log plot of relative concentrations of derivatized CA at 4 °C (blue) and 25 °C (green).

Considering the structural difference between the CAI and CA, it was observed that the chromophore is also unstable over time, following a different kinetic mechanism to the untreated CA. Statistical analysis with 95% of confidence (r^2^ = 0.998) indicated that solely temperature had a significant effect on the final concentration of the analyzed CAI, showing a typical Arrhenius-type behavior. Time courses for CAI with initial concentrations (CAI_o_) of 0.636 mM and 0.310 mM at 4 and 25 °C exhibited higher degradation rates than those for CA. In this case, the CAI showed linear dependency on concentration as it can be observed in the semi-log plot of relative concentrations for the CAI analyzed at different times (Figure 8), therefore, the degradation rate of the derivatized CA exhibited a first-order kinetics.

The calculated kinetic rate constants were 0.020 h^−1^ (r^2^ = 0.989) and 0.039 h^−1^ (r^2^ = 0.994) at 4 °C and 25 °C. Both values are closer to those calculated for the irreversible step of reaction in CA degradation at the same temperatures (Table 2). Regarding the Arrhenius parameters (r^2^ = 0.997), the calculated E_a_ was 21.85 kJ/mol, which is also close to the calculated value for the irreversible step for CA, the corresponding frequency factor (A) was 262.43 h^−1^. The trend of the time course of degradation for the CAI does not suggest an equilibrium process as for the case of CA, possibly due to the structural chemical modification; other degradation products are formed following a different mechanism. The results indicate a loss of 56% of CAI complex during the first 24 h, reaching a maximum of 82% at 45h and 25 °C. Usually, HPLC autosamplers provide samples cooling at 4 °C. However, at this temperature, the rate of degradation is also significant showing a 40% decay in 24 h and a maximum of 60% in 46 h. As expected, the degradation rate constant and hence, the half-life of the chromophore are independent from the initial CA concentration. The half-lives of the complex were 34.5 h and 17.8 h at 4 °C and 25 °C, respectively. Nevertheless, the degradation process is slowed down by cooling down the solution at 4°C; 10% of the CAI formed is lost in 5 h. These results suggest that it is convenient to spend a short time between sample derivatization and its injection in the HPLC column for assuring accurate quantification of CA, even if the sampler is cooled to 4 °C.

**Figure 9:**
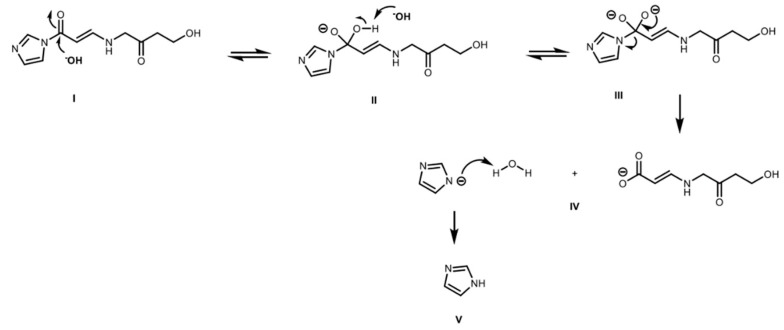
Base catalyzed hydrolysis of CAI: (I) Nucleophilic attack of hydroxyl to carbonyl group of CAI; (II) hydroxyl deprotonation and protonation of tertiary nitrogen; (III–IV) configuration of carboxyl group and elimination of imidazole ring; and (V) protonation of imidazole.

**Figure 10:**
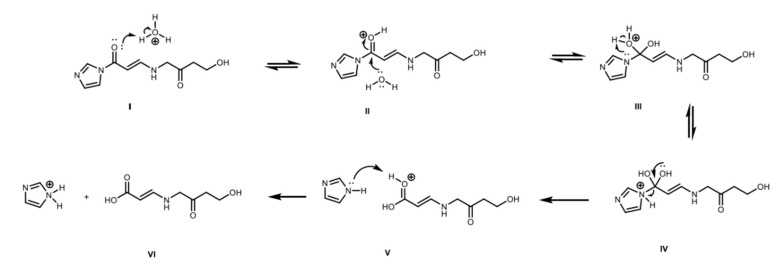
Acid catalyzed hydrolysis of CAI: (I) Protonation of carbonyl group of CAI; (II) Nucleophilic attack of water to carbonyl group of CAI; (III) water deprotonation; (IV) protonation of tertiary nitrogen and configuration or carboxyl group; (V) elimination of imidazole ring; and (VI) deprotonation of the carbonyl group and protonation of imidazole.

Since the chemical structure of the CA molecule is modified in the derivatization procedure, a different mechanism of reaction might be operating in the degradation of the chromophore. N-alkyl-imidazoles are hydrolytically unstable and they react under water-catalyzed, base-catalyzed (Figure 9), or acid-catalyzed (Figure 10) reaction mechanisms [33,34]. Therefore, it is probable that the degradation of the chromophore CAI occurs under one of those mechanisms depending on pH condition. CA is commonly produced and analyzed at slightly acidic conditions, hence, the degradation of the chromophore CAI would follow the mechanism presented in Figure 10. The irreversible hydrolysis of the complex might lead to underestimations of CA concentration of samples and/or overestimation of CA degradation rates due to the waiting time of the derivatized samples until the analyses are performed. 

## 3. Materials and Methods

*S. clavuligerus* DSM 41826 cryo-preserved at −80 °C in glycerol solution (16.7% *v*/*v*) was inoculated for activation in seed medium as described by Roubos et al. [20]. Batch fermentations were carried out in a 15 L stirred tank bioreactor (Techfors S, Infors AG, Bottmingen, Switzerland) operated at 5 L filling volume. Chemically defined media, composed as follows, were used [35]: glycerol (9.3 g/L), K_2_HPO_4_ (0.8 g/L), (NH_4_)_2_SO_4_ (1.26 g/L), monosodium glutamate (9.8 g/L), FeSO_4_·7H_2_O (0.18 g/L), MgSO_4_·7H_2_O (0.72 g/L) and trace element solution (1.44 mL). Trace elements solution contained: H_2_SO_4_ (20.4 g/L), monosodium citrate·1H_2_O (50 g/L), ZnSO_4_·7H_2_O (16.75 g/L), CuSO_4_·5H_2_O (2.5 g/L), MnCl_2_·4H_2_O (1.5 g/L), H_3_BO_3_ (2 g/L), and Na_2_MoO_4_·2H_2_O (2 g/L). Antifoam 204 was used at a concentration of 1:1000 *v*/*v*, pH was controlled at 6.8 by using NaOH 4M and HCl 4M. Aeration was provided at 0.6 VVM and temperature was controlled in 28 °C. 

Two samples (50 mL) of fermentation broth were withdrawn at 36 h of cultivation coinciding with phosphate limitation and exponential phase of growth, both conditions leading to the highest specific CA production and metabolic activity of the strain. Biomass and particulate material were separated by centrifugation at 12000 rpm and filtration using 0.2 µm pore size filters. Supernatants containing CA were adjusted to pH 6.8 and then vortexed and divided in 2 mL aliquots in Eppendorf tubes, according to the experimental design. Dilutions (1:2 and 1:5) were also prepared; finally, samples were divided into four groups and stored at the corresponding exposition temperatures. 

A factorial experimental design was proposed; concentration and temperature were defined as factors varying at three and four levels, respectively. The concentration of the supernatant was set as the highest level; dilutions 1:2 and 1:5 were set as the medium and low levels, respectively. Twelve experimental runs were performed by duplicate. Supernatant samples were stored at −80 °C, −20 °C, 4 °C, and 25 °C, respectively, for 43 h. Supernatant samples were withdrawn at 3.1 h, 5.4 h, 18.3 h, 31.0 h, and 42.1 h of storage, derivatized with imidazole solution 20% during 30 min at 30 °C and 800 rpm in a mixing block and immediately analyzed by HPLC. To test the degradation profile at a higher CA concentration than that of the experimental design, an additional duplicate run of supernatant samples with higher CA content (127 mg/L) from a different batch produced with identical medium and conditions was also exposed to the referred temperatures and treated as previously indicated. 

For the study of CAI stability, a 2-squared factorial design with duplicates was used. CA samples from fermentation broth with initial concentration of 127.0 mg/L (0.636 mM) and 61.7 mg/L (0.310 mM) were treated as previously described and derivatized. The derivatized samples were stored at 4 °C and 25 °C, aliquots were withdrawn at five different times in a time span of 46 h and analyzed by HPLC.

All the analyses of samples were carried out in an HPLC equipped with a DAD detector (1200 Series, Agilent Technologies, Waldbronn, Germany), using a Zorbax Eclipse XDB-C-18 chromatographic column (Agilent Technologies, Waldbronn, Germany) and a C-18 guard column (Phenomenex®, Aschaffenburg, Germany). The quantification of samples was carried out according to the gradient method described by Ramirez-Malule et al. [36]. 

The statistical analysis of the data for 95% of confidence was performed in Statgraphics Centurion XVII (Statgraphics Technologies, Inc. The Plains, VA, USA). Kinetic parameters were calculated by linear regression of experimental data with the least squares method; consistency was checked by determination of correlation coefficient (r^2^) and residual analysis. 

## 4. Conclusions

The kinetics of degradation of CA produced by *S. clavuligerus* DSM 41826 in a chemical defined medium was satisfactorily represented by two reaction models: one equilibrium reaction for intermediate formation and one irreversible first-order reaction for the degradation product formation. The equilibrium and irreversible reaction constants increased in parallel with temperature following the Arrhenius behavior. The proposed model showed a better fit with the experimental points than the traditional irreversible first-order model. Calculated NRMSE values at different temperatures and concentrations were less than 5% in all cases. 

The samples of CA in fermentation broth exhibited a fast decline of concentration during the first 5 h followed by a slower but stable reaction rate in the subsequent hours, which had been also observed in previous works. The reaction rate of degradation is dependent on several factors like pH, medium composition and temperature, which explains the high variability in the values of constant rates available in literature. The degradation rate at −80 °C is almost null, thus, this condition is appropriate for long-term storage of supernatants and stock solutions. At −20 °C the degradation rate in the time range explored is rather significant, but this condition is not expected to remain during a long time, since crystallization of the solution will avoid the progress of the reaction. 

Although the proposed model simplified the decomposition of CA to only one intermediate product and one decomposition product, it allowed to predict the CA concentration pattern in a rather reliable manner. The model is also able to account for the change in the reaction rate depending on the initial CA concentrations and supports the hypothesis of degradation as consequence of a susceptibility to nucleophilic attacks at specific points of the molecule, which also might coincide with hypothetical enzyme catalyzed reactions at specific conditions. 

Finally, the CAI chromophore, commonly used for spectrophotometric and HPLC analysis of CA samples, exhibited lower stability in time than the CA itself, possibly due to the susceptibility of N-alkyl-imidazoles to hydrolysis in aqueous solutions. Thus, a short time span is recommended between the derivatization of CA and the chromatographic analysis. For more reliable analysis of CA samples, after derivatization, the substance shall be conserved at 4 °C until the quantification is performed. Additionally, a temperature of −80 °C is recommended for long time storage of CA samples in order to avoid a significant loss of the product. 

## Figures and Tables

**Table 1:** Observed reaction rate constants for t < 5.5 h (k_obs,1_) and t > 5.5 h (k_obs,2_) at −80, −20, 4 and 25 °C.

Temperature	t < 5.5 h		t > 5.5 h	
	k_obs,1_ (h^−1^)	r^2^	k_obs,2_ (h^−1^)	r^2^
−80 °C	0.0009	0.969	0.0005	0.924
−20 °C	0.0135	0.991	0.0034	0.992
4 °C	0.0241	0.996	0.0035	0.998
25 °C	0.0320	0.998	0.0047	0.997

**Table 2:** Kinetic constants for the Equilibrium-Irreversible reaction model of CA degradation.

Temperature	Equilibrium Constant K_eq_	Irreversible Rate Constant k_2_ (h^−1^)
−80 °C	0.018	0.0009
−20 °C	0.162	0.0135
4 °C	0.210	0.0241
25 °C	0.280	0.0320

**Table 3:** Normalized-root-mean-square error (NRMSE) for the kinetic model of CA degradation.

Concentration (mg/L)	Temperature (°C)	NRMSE (%)	Shelf Life (t_90_)
126.7	−80	1.87	5.1 months
126.7	−20	3.22	9.0 h
126.7	4	2.18	6.5 h
126.7	25	3.78	4.5 h
65.5	−80	1.95	9.8 months
65.5	−20	4.98	9.7 h
65.5	4	4.93	6.6 h
65.5	25	4.45	4.6 h
25.3	−80	1.92	2.1 years
25.3	−20	3.36	9.9 h
25.3	4	3.27	6.7 h
25.3	25	3.40	4.7 h
16.3	−80	3.89	3.1 years
16.3	−20	1.98	10 h
16.3	4	2.53	6.8 h
16.3	25	2.42	4.8 h
